# Exploring the effect of wilting on fermentation profiles and microbial community structure during ensiling and air exposure of king grass silage

**DOI:** 10.3389/fmicb.2022.971426

**Published:** 2022-09-08

**Authors:** Rong Chen, Mao Li, Jinsong Yang, Liwei Chen, Xuejuan Zi, Hanlin Zhou, Jun Tang

**Affiliations:** ^1^Hainan University, Haikou, Hainan, China; ^2^Tropical Crops Genetic Resources Institute, Chinese Academy of Tropical Agricultural Sciences, Haikou, Hainan, China

**Keywords:** king grass, silage, bacterial community, fungal community, aerobic exposure

## Abstract

In order to better understand the effect of wilting treatment on silage, we study analyzed the fermentation quality of unwilted (CK) and wilted (WT) king grass silage, and the dynamic changes of microorganisms in silage and aerobic exposure. After 30 days of silage, WT silage significantly reduced the pH of the silage (*p < 0.05*) and increased the contents of lactic acid and acetic acid (*p < 0.05*), but did not reduce the content of Ammonia-N (*p > 0.05*). Wilting treatment increased bacterial and fungal diversity during silage but decreased fungal diversity during aerobic exposure. The relative abundance of *Lactococcus* and *Enterococcus* in wilting silage increased. In the aerobic exposure stage, the relative abundance of *Klebsiella* decreased, but the relative abundance of *Enterobacter* increased in wilting treatment silage. In addition, the relative abundance of *Acinetobacter* and *Ignatzschineria* increased after 5 days of aerobic exposure. In contrast with unwilted silage, wilting treatment silage after aerobic exposure had no *Candida*, but the relative abundance of *Wickerhamomyces* increased. The results showed that wilting treatment could raise the silage quality of king grass. However, WT silage did not inhibit the reproduction of harmful microorganisms during aerobic exposure and did not significantly improve the aerobic stability of silage.

## Introduction

King grass (*Pennisetum purpureum* Schumacher × *Penicillium glaucum* (Linnaeus) R. Brown) is a kind of high-quality grass, which is suitable for tropical and subtropical climates and likes warm and humid climates (Zhao et al., 2019). King grass is a nutritionally rich and high-yielding forage grass, which can be harvested 5–8 times per year for 75–180 t, which makes it an important animal feed resource (Botero-Londono et al., 2021; Long et al., 2022). In addition, king grass can absorb heavy metal ions from soil to purify soil (Li et al., 2021), which is rich in lignocellulosic biomass and can also be used to produce ethanol (Cardona et al., 2016). The growth of king grass is seasonal. It grows exuberant in the summertime or rainy season, but it grows slowly and has a low yield in winter or dry season, which is easy to leads to the imbalance between supply and demand (Li et al., 2014). According to the growth characteristics of king grass, using appropriate methods to store it during the bloom season can better provide a sustainable supply for ruminants. Ensiling is a common method for long-term storage of forage to improve feed palatability (Zhang et al., 2019a). The fermentation quality of silage quality is closely related to the nature of raw materials and the succession of microbial communities (Zi et al., 2021). After aerobic exposure, some harmful aerobic microorganisms such as yeasts and molds in the silage will multiply rapidly with the increase in temperature and pH, leading to the deterioration of the silage (Da Silva et al., 2015). Zhang et al. (2019a) reported the dynamic changes of microorganisms during sugarcane silage and after aerobic exposure. It is important to understand fully these changes for regulating the quality of silage fermentation. At present, the research on king grass silage mainly focuses on the mixed fermentation with other forages and the effects of adding starter on its silage quality and bacterial community (Li et al., 2014, 2019b; Zhang et al., 2018). However, there are no reports on the dynamic changes of microorganisms during the silage of king grass, and the microbial succession in the silage process is still unclear. Therefore, it is important to study the microbial dynamic changes during king grass silage and aerobic exposure to control the quality of king grass silage.

The fermentation quality of silage is influenced by many factors, among which raw material moisture and soluble carbohydrate content are important factors (Borreani et al., 2018). When the moisture content of the raw materials is too high, the growth of harmful microorganisms during silage is promoted, thereby reducing silage quality (Zhang et al., 2019b). Wilting is a conventional method to reduce the water content of raw materials. It has been shown that wilting treatment can inhibit the propagation of undesirable microorganisms and improves the quality of silage (Liu et al., 2011; Nishino et al., 2012; Wang et al., 2018). Guo et al. (2021) study showed that the relative abundance of *Lactobacillus* increased, while the relative abundances of *Enterobacter* and *Pseudomonas* decreased in paper mulberry silage after wilting treatment, and the results indicated that wilting treatment could improve paper mulberry silage quality. However, the quality of direct silage of king grass is not ideal because the dry matter content and soluble carbohydrates were low (Li et al., 2014; Zi et al., 2021). At present, there have been no reports on the microbial changes of wilted king grass silage. In addition, the effect of wilting on the quality, bacterial community, and fungal community of king grass silage is not clear.

The purpose of this study was to study the dynamic changes of bacterial and fungal communities of unwilted and wilted king grass during silage and aerobic exposure. It provides a theoretical basis for the structure of the king grass microbial strain bank and better improves the quality of king grass silage.

## Materials and methods

### Silage preparation

King grass (Reyan No. 4) was planted in the experimental base of the Chinese Academy of Tropical Agricultural Sciences (109°58′E, 19°52′N). Harvest the half-a-year vegetative king grass with strong growth and no diseases and pests as the experimental raw material. Select the period time without precipitation for 7 consecutive days for the sample, and the sampling time is the afternoon of November 2, 2020. All samples were taken from the same test field. After sampling, immediately put it into a sterile bag and take it back to the laboratory. The king grass is cut into small pieces of about 2 cm by a grass chopper (Donghong No. 1, Donghong Mechanical Equipment Co., Ltd., China). King grass was evenly divided into two parts, a part of king grass is silage directly without wilting treatment (CK). Another part of king grass is wilted until the water content is 63% (Wither at 30°C for 12 h), and then silage (WT). Before silage, a portion of each of the two groups was used for microbial diversity analysis. The unwilted and wilted king grass are packed in vacuum packaging bags (30 cm × 20 cm; Guo Zhong Packaging Co., Ltd., Haikou, China) according to 200 g per bag. The vacuum packaging machine (Xiangshan brand intelligent vacuum packaging machine; Xiangshan Tea Machinery Factory, Anxi County, Fujian Province, China) vacuums and stores it at a normal temperature of 30°C. A total of 60 bags (two treatment groups × six silage times × four aerobic exposure times × three repetitions). Samples were taken on days 1, 3, 5, 7, 14, and 30 of the fermentation process, and 1, 3, 5, and 7 after aerobic exposure. A part of the sample was used for microbial diversity analysis and a part of the sample was used for organic acid, pH, and Ammonia-N determination.

### Silage fermentation analysis

Place 25 g of the sample into a 250 ml volumetric flask containing 225 ml of sterile water and shake at 30°C for 2 h. After preliminary treatment, 40 ml of the samples were placed in 50 ml centrifuge tubes and centrifuged at 4°C, 10,000 × *g* for 15 min (Dong et al., 2020; Zi et al., 2021). After centrifugation, the supernatant was retained and the pH was measured using a pH meter (Starter 2,100; Ohaus Instruments Co. Ltd., Shanghai, China), organic acids (lactic acid, acetic acid, propionic acid, and butyric acid), and ammonia nitrogen determination, and the precipitate was retained at −80°C for microbial diversity analysis. The retained supernatant was filtered through a 0.22 μM filtration one part was assayed for pH with a pH glass electrode pH meter and one part was analyzed for lactic, acetic, propionic, butyric acid using an Agilent 1,200 HPLC (column: Athena C18-WP; eluent: 3 mM HClO_4_; flow rate:1 ml/min; temperature: 45°C; UVD:210 nm). Determination of ammonia-N content using a phenol-hypo-chlorite colorimetric method (Broderick and Kang, 1980).

### King grass silage microbial analysis

#### DNA extraction

Unwilted and wilted raw materials and silage from each ensiling period and aerobic exposure period were used for microbial community analysis. Briefly, 40 ml to 50 ml centrifuge tubes were taken from each bag of the treated filtrate, centrifuged at 10,000 r/m for 10 min at 4°C to remove the supernatant, and the precipitate was collected. Microbial extraction is carried out with reference to Zi et al. (2021) method.

#### PCR amplification and sequencing

The V3–V4 hypervariable region of bacterial 16S rRNA was amplified by primers 338F (5′-ACTCCTACGGGAGGCAGCAG-3′) and 806R (5′-GGACTACHVGGGTWTCTAAT-3′). Bacterial 16S amplification was performed as described by Tian et al. (2017). Fungal ITS amplicon sequencing used BITS (5′-NNNNNNNNCTACCTGCGGARGGA-TCA-3′) and B58S3 (5′-GAGATCCRTTGYTRAAAGTT-3′) universal primers. Fungal ITS-1 amplification was conducted according to the description of Romero et al. (2017). Illumina Miseq 2500 platform (Illumina, Inc., San Diego, CA, United States) was used for sequencing. UCHIME algorithm is used to obtain valid data. Calculate Shannon, Chao1, ACE, and Good coverage by QIIME (version 2.15.3). The sequencing data of this study has been submitted to the NCBI database, and the Bioproject registration number is PRJNA76755.

#### Statistical analysis

The SPSS2016 software was used to conduct a one-way analysis of variance (ANOVA) of each index of king grass silage, and the general linear model (GLM) was used to conduct a two-way analysis of variance on treatment method, silage duration, and their interaction. Comparisons at the 5% significance level were made using multiple comparison tests of Duncan. The online platform (Illumina hiseq sequencing platform) was used to analyze sequencing data for the bacterial and fungal communities.

## Results

### Fermentation profiles of king grass silage during ensiling and aerobic exposure

The fermentation quality of CK silage and WT silage are shown in Table 1. The pH value of silage decreased gradually with the prolonged silage time. The contents of lactic acid and acetic acid increased and decreased during silage, but showed an overall increasing trend. Propionic acid and butyric acid were not detected in CK silage and WT silage. After 30 days of silage, WT silage exhibited significantly lower pH than CK silage (*p*<0.01). After 30 days of silage, the contents of lactic and acetic acids in WT silage were significantly higher than in CK silage (*p* < 0.01). In addition, the present study found that ammonia nitrogen (Ammonia-N) content was low in king grass silage. After 30 days of silage, there was no notable difference in ammonia-N content between CK silage and WT silage (*p* > 0.05).

**Table 1:** Fermentation characteristics of king grass silage during silage.

Item	Treatment	Ensiling days						SEM	*p*-Value		
		1	3	5	7	14	30		D	T	D*T
pH	CK	5.30^aA^	4.95^bA^	4.89^bA^	4.81^cA^	4.64^dA^	4.54^eA^	0.06	<0.01	<0.01	<0.01
	WT	4.81^aB^	4.51^bB^	4.49^bB^	4.48^bcB^	4.43^cB^	4.32^dB^	0.04			
Lactic acid (g/kg DM)	CK	0.13^aA^	0.52^bA^	0.36^bA^	0.88^cA^	3.90^dA^	2.61^eA^	0.34	<0.01	<0.01	<0.01
	WT	0.77^aB^	1.42^bB^	3.63^cB^	1.73^dB^	3.45^eB^	4.07^fB^	0.30			
Acetic acid (g/ kg DM)	CK	4.87^aA^	7.01^bA^	6.58^cA^	9.99^dA^	15.84^eA^	14.86^fA^	1.01	<0.01	<0.01	<0.01
	WT	4.01^aB^	9.79^bB^	14.5^cB^	10.11^bA^	13.29^dB^	16.73^eB^	0.99			
Propionic acid (g/ kg DM)	CK	ND	ND	ND	ND	ND	ND	–	–	–	–
	WT	ND	ND	ND	ND	ND	ND				
Butyric acid (g/ kg DM)	CK	ND	ND	ND	ND	ND	ND	–	–	–	–
	WT	ND	ND	ND	ND	ND	ND				
Ammonia-N (g/ kg DM)	CK	0.038^aA^	0.042^aA^	0.025^bA^	0.027^bA^	0.020^bA^	0.025^bA^	0.0029	<0.01	<0.01	<0.01
	WT	0.009^aB^	0.041^bA^	0.028^cA^	0.021^cdB^	0.027^cdB^	0.020^dA^	0.0029			

CK, unwilted silage; WT, wilting silage; ND, not detected; DM, dry matter; D, ensiling days; T, treatment; D*T, the interaction of ensiling days and treatment; SEM, standard error of means. Means with different letters in the same row (a–e) or column (A–B) differ (*p* < 0.05).

As shown in Table 2, although the pH of king grass silage increased with the extension of aerobic exposure time, the pH of the WT silage was significantly lower than that of the CK silage (P<0.01). In addition, the pH values of CK silage and WT silage increased slowly 3 days before aerobic exposure. However, after being exposed to the air for 5 days, the pH increased rapidly. After being exposed to the air for 7 days, the content of lactic acid in WT silage was prominent higher than in CK silage (*p* < 0.01), while the content of acetic acid was significantly lower than in CK silage (*p* < 0.01). After being exposed to the air for 7 days, the Ammonia-N content in CK silage was prominent higher than that in WT silage (*p* < 0.01).

**Table 2:** Fermentation characteristics of king grass silage after aerobic exposure.

Item	Treatment	Aerobic exposure days				SEM	*p*-Value		
		1	3	5	7		D	T	D*T
pH	CK	4.65^aA^	4.96^bA^	5.76^cA^	6.50^dA^	0.22	< 0.01	< 0.01	< 0.01
	WT	4.48^aB^	4.75^bB^	5.39^cB^	6.05^dB^	0.18			
Lactic acid (g/kg DM)	CK	2.48^abA^	2.46^baA^	1.73^cA^	0.93^dA^	0.19	< 0.01	< 0.01	< 0.01
	WT	3.67^abB^	3.58^baB^	3.03^cB^	1.70^dB^	0.24			
Acetic acid (g/kg DM)	CK	11.30^aA^	18.91^bA^	13.33^cA^	12.20^dA^	0.90	< 0.01	< 0.01	< 0.01
	WT	19.46^aB^	15.28^bB^	10.39^cB^	9.96^cB^	1.17			
Propionic acid (g/kg DM)	CK	ND	ND	ND	ND	–	–	–	–
–	WT	ND	ND	ND	ND				
Butyric acid (g kg DM)	CK	ND	ND	ND	ND	–	–	–	–
–	WT	ND	ND	ND	ND				
Ammonia-N (g/kg DM)	CK	0.028^aA^	0.054^bA^	0.117^cA^	0.251^dA^	0.0261	< 0.01	< 0.01	< 0.01
	WT	0.037^aB^	0.054^bA^	0.069^cB^	0.195^dB^	0.0189			

CK, unwilted silage; WT, wilting silage; ND, not detected; DM, dry matter; D, ensiling days; T, treatment; D*T, the interaction of ensiling days and treatment; SEM, standard error of means. Means with different letters in the same row (a–e) or column (A–B) differ (*p* < 0.05).

### Bacterial diversity of king grass silage during silage and aerobic exposure

Table 3 is Alpha diversity of bacteria in king grass ensiling process and after exposure to air. As presented in Table 3, Good’s coverage value was about 1, which illustrated that the sample assay covered most of the bacteria, reflecting the real situation of the bacteria in the sample. It can be seen from the indexes of Outs and Chao 1 that with the extension of silage time, the bacterial colony richness in CK silage and WT silage is relatively stable and has no obvious change. Compared with CK silage, the bacterial community richness and diversity of WT silage were lower than CK silage.

**Table 3:** Alpha diversity of bacteria in king grass ensiling process and after exposure to air.

Sample ID	Reads	Outs	Shannon	Chao 1	ACE	Coverage
CK0	77,630	270	1.922	291.033	287.341	1
CK1	77,624	283	2.588	297.057	291.122	1
CK3	78,247	264	1.167	304.825	304.283	1
CK5	77,664	256	1.513	288.623	287.934	1
CK7	77,783	304	3.824	317.333	313.876	1
CK14	77,954	301	2.856	311.060	307.960	1
CK30	77,741	299	1.903	320.205	310.667	1
CKE1	69,470	304	1.944	304.410	319.376	1
CKE3	77,907	219	0.906	274.940	272.001	1
CKE5	77,925	191	0.687	247.383	282.158	1
CKE7	72,065	172	2.441	252.948	317.531	1
WT0	76,187	231	2.423	263.514	263.764	1
WT1	77,495	274	3.488	295.282	290.132	1
WT3	78,294	266	2.540	278.464	274.818	1
WT5	78,032	223	1.718	273.448	273.605	1
WT7	75,549	260	2.684	276.558	276.621	1
WT14	78,334	249	2.259	281.010	272.521	1
WT30	77,633	264	2.376	280.627	276.536	1
WTE1	77,019	236	2.169	293.0382	331.132	1
WTE3	78.053	263	1.723	291.339	286.400	1
WTE5	74,377	196	2.252	277.733	265.594	1
WTE7	77,915	195	1.660	246.975	244.063	1

CK, unwilted silage; CKE, unwilted silage aerobic exposure; WT, Wilting silage; WTE, Wilting silage aerobic exposure. Arabic numerals indicate time spent silage.

Under aerobic exposure, although the bacterial colony richness in CK silage and WT silage decreased with time, the bacterial richness of CK silage was lower than that of WT silage after being exposed to the air for 7 days. Aerobic exposure of silage on days 3 and 5, the Shannon index was higher in WT silage than in CK silage. However, on day 7 of exposure to air, the Shannon index of WT silage was noteworthy lower than CK silage.

### Bacterial community dynamics during king grass silage and aerobic exposure

**Figure 1:**
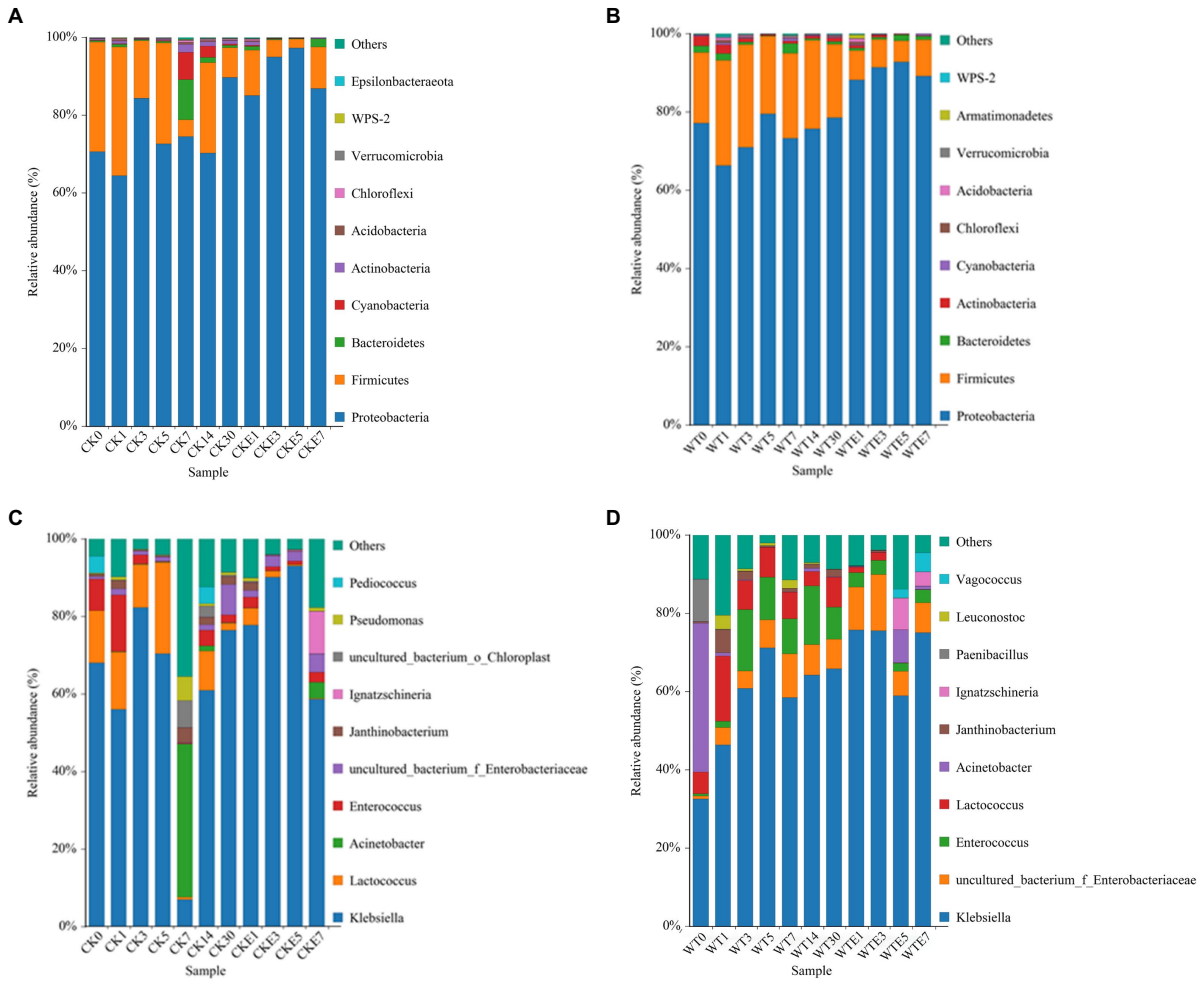
Relative abundance of bacterial community during ensiling and aerobic exposure of King grass silage. **(A,C)** show the relative abundance at phylum and genus level for CK, respectively, while **(B,D)** show the relative abundance at phylum and genus level for WT, respectively. CK, unwilted silage; CKE, unwilted silage aerobic exposure; WT, wilting silage; WTE, wilting silage aerobic exposure; Arabic numerals indicate the time of silage.

The bacterial dynamic changes during the king grass ensiling period and aerobic exposure are presented in Figure 1. Where Figures 1A,B are the bacterial community dynamics at the phylum level. Before silage, the dominant bacteria in king grass were mainly *Proteobacteria* and *Firmicutes*. Compared with unwilted, after wilted treatment, the relative abundance of *Proteobacteria* (70.56 ~ 77.19%) increased, while that of *Firmicutes* (28.22 ~ 17.96%) decreased. The relative abundance of *Cyanobacteria* was higher in CK silage (6.99 and 3.21%, respectively) on days 7 and 14 of ensiling in contrast with WT silage (0.63 and 0.17%, respectively). In addition, *Bacteroidetes* (10.21%) and *Actinobacteria* (2.11%) were more abundant in CK silage on the 7 day of ensiling. On day 30 of ensiling, the relative abundance of *Proteobacteria* in WT silage (78.36%) was lower than that of CK silage (89.73%), but the relative abundance of *Firmicutes* was higher in WT silage (18.87%) than in CK silage (7.61%). *Proteobacteria* remain the dominant phylum in CK silage and WT silage aerobic exposure. The relative abundance of *Proteobacteria* increased, while that of *Firmicutes* decreased in CK silage and WT silage 5 days before aerobic exposure. However, on day 7 of exposure to air, the relative abundance of *Proteobacteria* in CK silage and WT silage decreased, while the relative abundance of *Firmicutes* increased. Totality, *Proteobacteria* and *Firmicutes* were the two phyla with the highest relative abundance in king grass silage at any time of ensiling and aerobic exposure.

**Figure 2:**
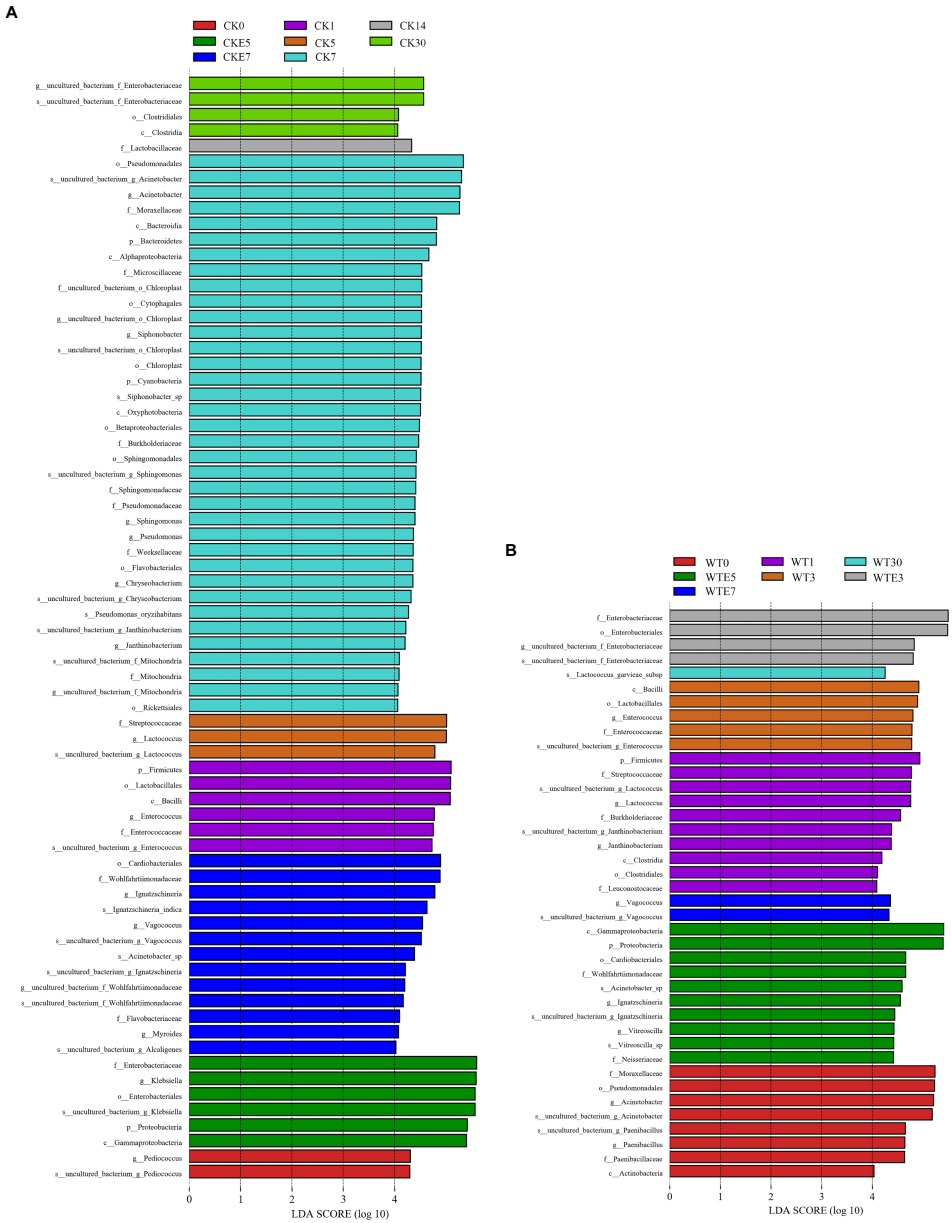
Comparison of bacterial changes during ensiling and aerobic exposure using LEfSe analysis. (**A**, CK group; **B**, WT group; CK, unwilted silage; CKE, unwilted silage aerobic exposure; WT, Wilting silage; WTE, Wilting silage aerobic exposure; Arabic numerals indicate the time of silage).

Figures 1C,D. show the dynamic changes of bacterial genus level. In this study, lactic acid bacteria present in both unwilted and wilted king grass before silage were mainly *Lactococcus* and *Enterococcus*. In addition, *Pediococcus* was present in unwilted king grass, whereas *Leuconostoc* was present in wilting treatment king grass. During the ensiling, regardless of CK silage (68.02% ~ 76.43%) or WT silage (32.55% ~ 65.78%), *Klebsiella* was the dominant bacterial genus during the silage of king grass. In contrast with CK silage, on the 3 and 5 days of ensiling, the relative abundance of *Lactococcus* was lower and that of *Enterococcus* was higher in the WT silage. On days 5 to 7 of silage, the relative abundance of *Klebsiella* decreased from 70.38 to 6.99%, the relative abundance of *Lactococcus* decreased from 23.54 to 0.63%, and the relative abundance of *Acinetobacter* increased from 0.17 to 39.51% in CK silage. WT silage (7.67 and 8.19%, respectively) showed higher abundances of *Lactococcus* and *Enterococcus* than CK silage (1.83 and 2.03%, respectively) at day 30 of ensiling. In addition, after 30 days of ensiled, *Lactococcus* and *Enterobacter* were the most different species between direct silage and withered silage (Figure 2). The relative abundance of *Enterobacteriaceae* in CK silage (7.87%) and WT silage (7.57%) did not change much after 30 days of ensiled. After aerobic exposure, *Klebsiella* remained the dominant genus in both CK silage and WT silage. On day 1 of aerobic exposure, the relative abundance of *Lactococcus* increased from 2.03 to 2.78% and that of *Enterococcus* increased from 1.83 to 4.39% in CK silage. In contrast, the relative abundance of *Lactococcus* decreased from 7.67 to 1.36% and the relative abundance of *Enterococcus* decreased from 8.19 to 3.69% in WT silages on day 1 of aerobic exposure. *Lactococcus* abundance decreased rapidly in both CK silage and WT silage from days 3 to 7 of aerobic exposure. The relative abundance of *Lactococcus* decreased from 4.39 to 0.19% in the CK silage and from 2 to 0.09% in the WT silage. The abundance of *Enterobacteriaceae* in WT silage increased from 7.58 to 14.37% on days 1 to 3 of aerobic exposure. The relative abundance of *Acinetobacter* increased in silage after being exposed to the air for 5 days. On days 5 and 7 of aerobic exposure, the relative abundance of *Acinetobacter* in CK silage and WT silage was significantly different (Figure 2).

### Fungal diversity of king grass silage during silage and aerobic exposure

Table 4 is Alpha diversity of fungal in king grass ensiling process and after exposure to air. It can be seen from Table 4 that the CK silage Chao1 and ACE indicators showed a decreasing trend during days 1–30 of ensiling, while WT silage Chao1 and ACE indicators presented an increasing trend. After 30 days of silage, the Chao1, ACE, and Shannon indices were higher in WT silage than in CK silage, indicating that wilt treatment increased the king grass fungal richness and diversity.

**Table 4:** Alpha diversity of fungal in king grass ensiling process and after exposure to air.

Sample ID	Reads	Outs	Shannon	Chao 1	ACE	Coverage
CK0	78,165	529	6.995	820.528	1119.710	1
CK1	77,594	442	5.960	821.488	975.710	1
CK3	79,099	728	7.111	1033.652	1378.050	1
CK5	78,798	613	7.838	875.650	1376.560	1
CK7	78,886	710	7.621	925.211	991.060	1
CK14	78,792	434	6.998	687.630	857.490	1
CK30	78,091	381	6.482	678.629	882.720	1
CKE1	78,866	442	6.685	790.191	1191.050	1
CKE3	78,515	457	6.476	836.504	1176.540	1
CKE5	78,423	497	6.267	785.283	863.010	1
CKE7	78,871	484	4.540	867.323	1170.460	1
WT0	78,453	565	6.418	862.118	1114.250	1
WT1	78,734	622	7.534	955.138	1243.770	1
WT3	78,383	564	6.735	918.222	1218.200	1
WT5	78,760	586	6.793	951.396	1087.810	1
WT7	78,795	542	7.081	905.820	1196.810	1
WT14	78,946	541	7.034	915.974	1096.850	1
WT30	78,961	491	7.060	846.248	1201.850	1
WTE1	78,954	704	7.0.977	1058.347	1207.310	1
WTE3	78,939	478	6.774	864.324	983.400	1
WTE5	78,861	550	5.603	890.574	1151.290	1
WTE7	78,993	418	4.735	758.109	1134.040	1

CK, unwilted silage; CKE, unwilted silage aerobic exposure; WT, wilting silage; WTE, wilting silage aerobic exposure. Arabic numerals indicate time spent silage.

After aerobic exposure, the Chao1 and ACE indexes in the CK silage showed an increasing trend, while those in the WT silage showed a decreasing trend, indicating that the relative abundance of fungi decreased in the WT silage with aerobic exposure. After being exposed to the air for 7 days, fungal diversity was lower in WT dried silage than in CK silage.

### Fungal community dynamics during king grass silage and aerobic exposure

**Figure 3:**
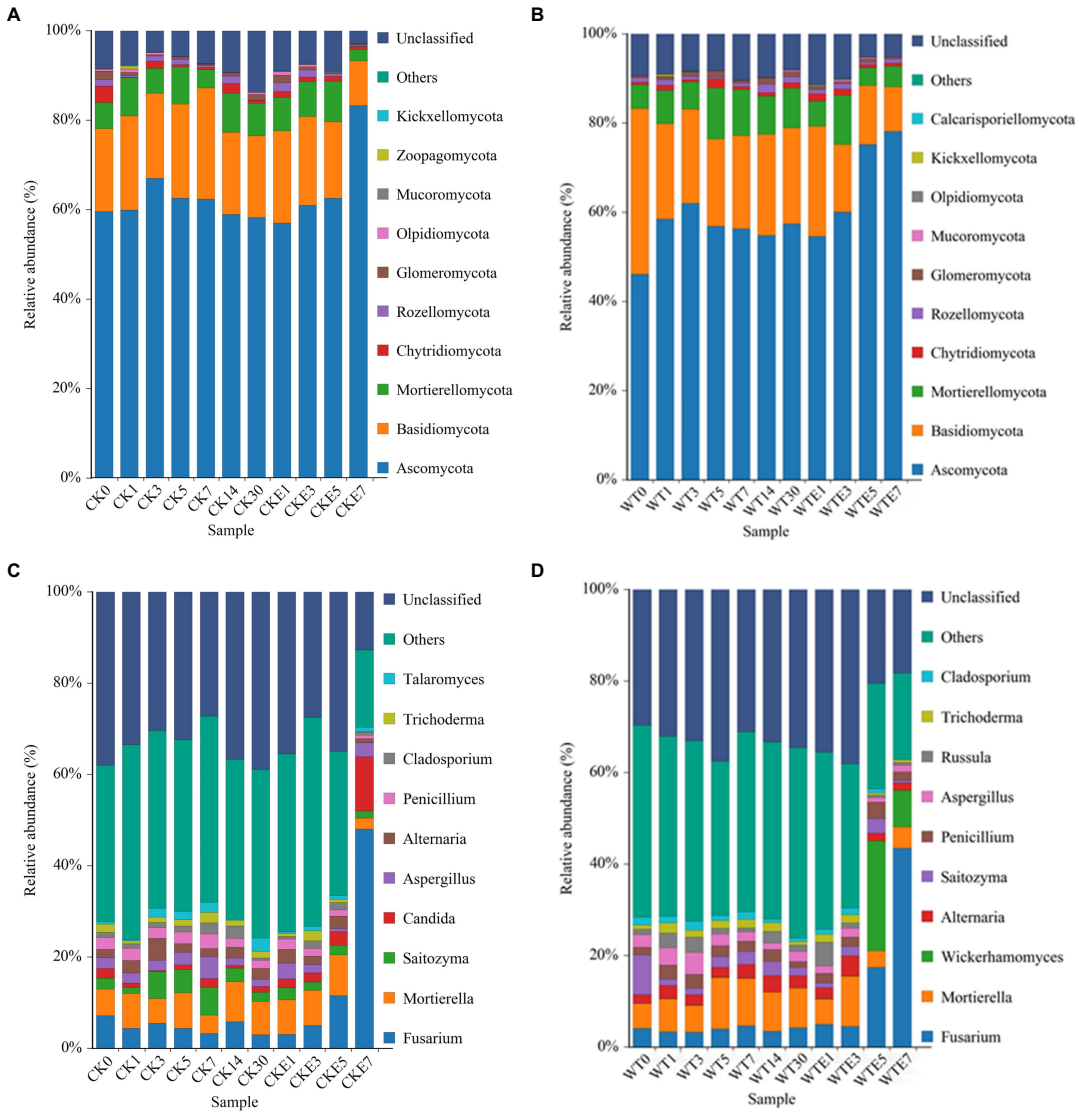
Relative abundance of fungal community during ensiling and aerobic exposure of King grass silage. **(A,C)** show the relative abundance at phylum and genus level for CK, respectively, while **(B,D)** show the relative abundance at phylum and genus level for WT, respectively. CK, unwilted silage; CKE, unwilted silage aerobic exposure; WT, wilting silage; WTE, wilting silage aerobic exposure; Arabic numerals indicate the time of silage.

**Figure 4:**
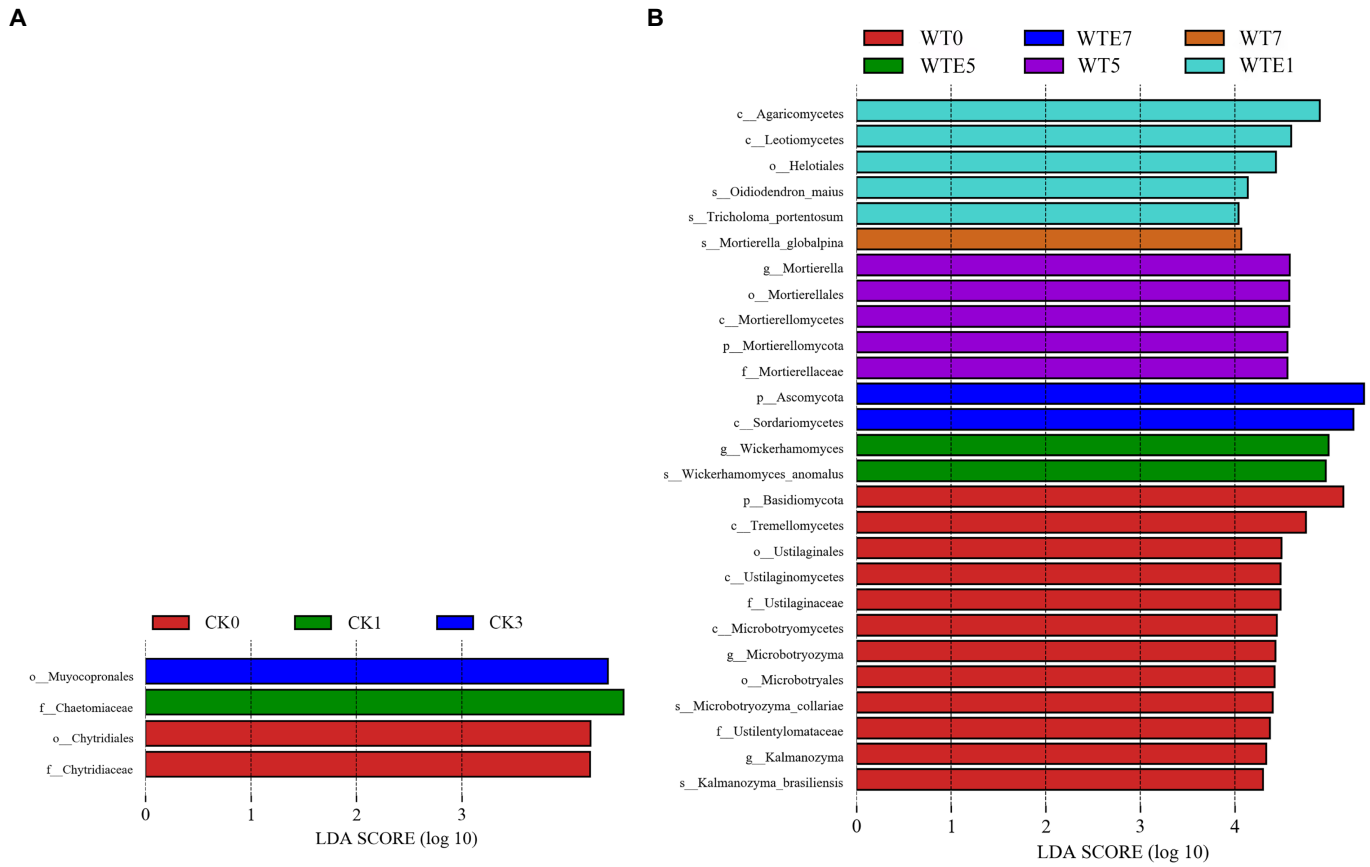
Comparison of fungal changes during ensiling and aerobic exposure using LEfSe analysis. (**A**, CK group; **B**, WT group; CK, unwilted silage; CKE, unwilted silage aerobic exposure; WT, wilting silage; WTE, wilting silage aerobic exposure; Arabic numerals indicate the time of silage.)

The fungal dynamic changes during the king grass ensiling period and aerobic exposure are presented in Figure 3. Where Figures 3A,B are the fungal community dynamics at the phylum level. Before silage, the main fungal phyla in unwilted king grass and wilted king grass are *Ascomycota*, *Basidiomycota* and *Mortierellomycota*. In addition, Figure 4 shows that there is a significant difference in the relative abundance of *Basidiomycota* between CK silage and WT silage. *Ascomycota* relative abundance was decreased while *Basidiomycota* relative abundance was increased in wilting (50.84 and 41.01%, respectively) king grass compared to unwilted (65.04 and 20.22%, respectively) king grass. *Ascomycota* and *Basidiomycota* were both the dominant phyla in CK silage and WT silage during ensiling. *Ascomycota* and *Basidiomycota* remained the dominant phyla in CK silage and WT silage after aerobic exposure. After being exposed to the air, the relative abundance of *Ascomycota* began to increase. After 7 days of aerobic exposure, the relative abundance of Ascomycota in CK silage increased from 67.61 to 85.58%, and that in WT silage increased from 62.5 to 82.22%.

Figures 3C,D show the dynamic changes of the fungal genus level. *Fusarium*, *Mortierella*, *Aspergillus*, *Penicillium*, *Saitozyma*, and *Alternaria* were found in CK silage and WT silage during ensiling. In addition, *Candida* was also identified in CK silage (2.1%), however, there was no Candida in the WT silage. The relative abundance of *Wickerhamomyces* in WT silage increased to 24.09% on day 5 of aerobic exposure, but decreased to 7.98% on day 7 of aerobic exposure. *Wickerhamomyces* were not detected in CK silage. After exposure to air, the richness of the *Fusarium* in CK silage and WT silage was raised. On the 7th day of aerobic exposure, the relative abundance of *Fusarium* in CK silage increased from 2.94 to 48.03%, and that in WT silage increased from 4.2 to 43.48%. After exposure to air for 7 days, the relative abundance of *Candida* in CK silage increased from 1.27 to 11.92%. After 7 days of aerobic exposure, the relative abundance of *Penicillium* in CK silage decreased from 1.85 to 0.77%, while the relative abundance of *Penicillium* in WT silage increased from 1.3 to 1.84%.

## Discussion

In this study, wilting treatment reduced silage pH, raised the contents of lactic acid and acetic acid, and improved silage fermentation quality. pH and organic acid are important indexes to evaluate silage quality. In contrast with CK silage, after 30 days of silage, the pH of WT silage is lower, and the contents of lactic acid and acetic acid are higher, which indicates that the quality of WT silage is better. Besides, the content of acetic acid in king grass silage was higher than that of lactic acid, indicating that king grass silage was a combination of lactic acid fermentation and acetic acid fermentation, but acetic acid fermentation was dominant, which may also be the reason for the high pH of silage. Studies have shown that acetic acid is the main organic acid in tropical forages (Nishino et al., 2012; Li et al., 2019a). In addition, high concentrations of lactic acid and acetic acid contribute to silage fermentation, which may be because high concentrations of lactic acid and acetic acid inhibit the multiplication of unwelcome microorganisms. Ammonia-N is the product of protein hydrolysis and an important index for assessing the quality of silage (Li et al., 2018). The protein in silage will be hydrolyzed into Ammonia-N and other substances, reducing the absorption and utilization of protein by ruminants (Kung et al., 2018). It was found that there was no notable difference in Ammonia-N content between CK silage and WT silage, indicating that wilting treatment did not affect the hydrolytic activity of the protease. After 5 days of aerobic exposure, the pH in the feed increased rapidly, indicating that the aerobic spoilage of silage was aggravated. Combined with the increase in the relative abundance of yeasts and molds after 5 days of aerobic exposure in Figure 3, it shows that wilting does not improve the aerobic stability of king grass silage.

During silage and aerobic exposure, *Proteobacteria* and *Firmicum* are the two phyla with high relative abundance, which is the same as other people’s research (Hu et al., 2018; Zhang et al., 2019a). On day 7 of silage, the richness of *Bacteroidetes* and *Actinobacteria* increased suddenly. The reason for this phenomenon is difficult to explain, so we need to conduct more in-depth research. Zi et al. (2021) showed that after 30 days of silage, the relative abundance of *Firmicutes* in king grass silage was higher than that of *Proteobacteria*, which was just the opposite in this study. It has been reported that with the increase in silage time, the dominant phylum will change from *Proteobacteria* to *Firmicutes* (Xu et al., 2017; Yuan et al., 2019). However, this transformation needs to be carried out in a low pH and anaerobic environment (Dong et al., 2019). In this study, the higher pH (> 4.3) of king grass silage, resulted in no shift from *Proteobacteria* to *Firmicutes*. This is consistent with studies by others (Long et al., 2022). After exposure to air, the relative abundance of *Proteobacteria* raised and that of *Firmicutes* decreased, which is consistent with the research of others (McGarvey et al., 2013; Liu et al., 2019). Lactic acid bacteria related to silage mainly include *Lactobacillus*, *Lactococcus*, *Pediococcus*, *Leuconostoc*, *Enterococcus*, etc. (Tohno et al., 2012). *Lactococcus* plays a key role in silage and has been widely used at present (Ni et al., 2018). *Lactococcus* begin to produce lactic acid early in silage, decreasing the pH of silage and providing an acidic environment for the growth of other lactic acid bacteria. Most studies showed that during the late stage of silage, *Lactococcus* would be replaced by *Lactobacilli* with better acid tolerance properties (Keshri et al., 2018; Yuan et al., 2019). Yang et al. (2016) research that *Lactobacillus* was the dominant species in fruit residue, could survive at a lower acid base, and produced more lactic acid. But in this study, after 30 days of silage, there was no *Lactobacillus* in silage and *Lactococcus* was still the dominant genus. The possible reason was that the pH of silage was higher, *Lactococcus* could still survive and play a significant role. Besides, *Enterococcus*, *Pediococcus*, and *Leuconostoc* also play a significant role in the early silage. Parvin et al. (2010) discover that *Enterococcus* existed in the wilted guinea grass silage compared to direct silage. Moreover, Ni et al. (2017) also reported that *Enterococcus* in withered alfalfa silage is the dominant genus. In this research, the richness of *Enterococcus* raised in silage after wilting treatment, is consistent with previous studies. *Klebsiella* is a facultative anaerobic bacterium and a harmful microorganism in silage (Lin et al., 2021). *Klebsiella* has been found in many silages, such as corn, tea, mulberry leaves, etc. (Hu et al., 2018; Wang et al., 2020; Lin et al., 2021). Studies have shown that pH < 4 can inhibit the growth of *Klebsiella* (Junges et al., 2017). In this study, *Klebsiella* has always been the dominant bacteria, which may be related to the pH > 4.3 of king grass silage, and too high pH not inhibit its growth. *Enterobacteriaceae* bacteria are generally undesirable bacteria in silage, which ferment lactic acid into acetic acid and other products (Santos et al., 2016). It has been shown that the occurrence of *Enterobacteriaceae* is associated with an acetic acid content, and the relative abundance of *Enterobacteriaceae* tends to be higher in silages with high acetic acid content (Nishino et al., 2012; Li et al., 2019a). This research further confirms this view. *Acinetobacter* is an aerobic bacterium that can be detected in diverse surroundings (Kampfer and Glaeser, 2012). Studies have shown that *Acinetobacter* does not exist in silage (Li and Nishino, 2011). However, Ogunade et al. (2017) found the presence of *Acinetobacter* in silage after 120 days of ECLB silage and speculated that the raised relative abundance of *Acinetobacte*r may be related to the raised acetic acid concentration. It has been shown that some *Acinetobacter* can survive when acetate is sufficient in the surrounding environment (Fuhs and Chen, 1975). In this study, given the increased relative abundance of *Acinetobacter* and higher-level WT of acetic acid in silage on day 7 of CK silage and WT silage, we speculate that a proportion of *Acinetobacter* species utilize acetic acid and thus survive the anaerobic phase of silage.

*Ascomycota* and *Basidiomycota* are the most abundant fungal phyla during silage and aerobic exposure, which is the same as other people’s research (Romero et al., 2017; Zhang et al., 2019a). *Fusarium*, *Aspergillus*, and *Penicillium* are common undesirable molds (Vu et al., 2019). *Fusarium* can produce a variety of mycotoxins, which affect the feeding safety of silages (Niderkorn et al., 2007). Myllykoski et al. (2009) reported that beef cattle cause their jejuna hemorrhagic syndrome after consuming a diet containing various mycotoxin-producing species including *Fusarium*, *Aspergillus*, and *Penicillium*. Yeast propagates rapidly after aerobic exposure, resulting in silage corruption. The most common yeast genera involved in silage aerobic spoilage are *Pichia*, *Candida*, and *Issatchenkia* (Liu et al., 2019; Zhang et al., 2019a). Yeasts consume sugars and organic acids, resulting in increased silage pH and temperature, extensive growth of other aerobic microorganisms, and finally complete deterioration of the silage by molds (Borreani and Tabacco, 2010). In the present study, the presence of *Candida* was detected only after being exposed to the air for 5 days from CK silage. *Candida* is a lactic acid assimilating yeast that raised aerobic spoilage of silage (Bai et al., 2020). Besides, the presence of *Wickerhamomyces* was detected after being exposed to the air for 5 days in WT silage, but fewer studies are currently available on *Wickerhamomyces* in silage. We speculate that *Wickerhamomyces* promotes the aerobic deterioration of king grass silage. In addition, *Fusarium* grew abundantly in both CK and WT silages after 5 days of aerobic exposure. The above results indicated that wilting could not inhibit the growth of harmful microorganisms after aerobic exposure, indicating that wilting did not improve the aerobic stability of king grass silage.

## Conclusion

The findings show that the wilting treatment significantly raised the content of lactic acid and acetic acid, and lowered the pH. The wilting treatment increased the bacterial and fungal diversity of silage. Besides, the wilting treatment increased the relative abundance of *Lactococcus* and *Enterococcus* in king grass silage. But the wilting treatment did not raise the aerobic stability of silage. After exposure to air, the relative abundance of *Lactococcus* decreased and that of *Acinetobacter* and *Ignatzschineria* increased. After being exposed to the air for 7 days, CK silage and WT ensiling fungal colony were dominated by *Fusarium*. *Candida* and *Wickerhamomyces* belong to the *Saccharomyces* and are important microorganisms in silage deterioration upon aerobic exposure.

## Data availability statement

The datasets presented in this study can be found in online repositories. The names of the repository/repositories and accession number(s) can be found at: https://submit.ncbi.nlm.nih.gov/PRJNA767755.

## Author contributions

RC, ML, and XZ carried out the experimental design. RC, ML, LC, and XZ performed the experiments. RC, ML, LC, JY, XZ, JT, and HZ analyzed the experimental data. RC wrote the first draft. ML and XZ revised the manuscript. All authors contributed to the article and approved the submitted version.

## Funding

This study was funded by the National Natural Science Foundation of China (Nos. 31860680 and 31960678), the Key Research and Development Program of Hainan Province (ZDYF2022XDNY153), and the Central Public-Interest Scientific Institution Basal Research Fund for Chinese Academy of Tropical Agricultural Sciences (1630032022011).

## Conflict of interest

The authors declare that the research was conducted in the absence of any commercial or financial relationships that could be construed as a potential conflict of interest.

## Publisher’s note

All claims expressed in this article are solely those of the authors and do not necessarily represent those of their affiliated organizations, or those of the publisher, the editors and the reviewers. Any product that may be evaluated in this article, or claim that may be made by its manufacturer, is not guaranteed or endorsed by the publisher.
